# Biological Activity of Hops (*Humulus lupulus* L.): Molecular Mechanisms and Significance for Human Health—A Review

**DOI:** 10.3390/nu18071056

**Published:** 2026-03-26

**Authors:** Łukasz Kogut, Czesław Puchalski, Julia Jastrzębska, Grzegorz Zaguła

**Affiliations:** 1 Department of Bioenergetics, Food Analysis and Microbiology, Institute of Food Technology and Nutrition, Faculty of Technology and Life Science, University of Rzeszów, Aleja Rejtana 16C, 35-959 Rzeszów, Poland; lukasz.kogut2@wp.pl (Ł.K.); cpuchal@ur.edu.pl (C.P.); 2 Medical Centre in Łańcut LLC, Ignacego Paderewskiego 5, 37-100 Łańcut, Poland; j.jastrzebska2105@gmail.com

**Keywords:** common hop, *Humulus lupulus* L., polyphenols, inflammation, oxidative stress, xanthohumol, metabolism, gut microbiota

## Abstract

**Introduction/Objective:** Common hop (*Humulus lupulus* L.) is a multi-component plant material that has been extensively studied for its antioxidant, anti-inflammatory, cardioprotective, metabolic, neuroprotective, immunomodulatory and anti-cancer properties. This review summarises current data on the molecular mechanisms of action of hop compounds, their therapeutic potential, metabolic interactions and biological significance, with particular emphasis on bioavailability, signalling pathways and organ-specific effects. **Methods:** A comprehensive literature review was conducted, covering in vitro and in vivo studies and available clinical trials analysing the biochemical activity, molecular targets and physiological effects of bioactive compounds in hops. Particular attention was paid to the regulation of oxidative stress, inflammatory signalling, mitochondrial function, metabolic pathways, interactions with the gut microbiota and their impact on the development of chronic diseases. **Results:** Bioactive compounds in hops modulate numerous key signalling pathways, including NF-κB, Nrf2, AMPK, MAPK, PPAR and PI3K/AKT/mTOR. They have been shown to reduce oxidative stress, inhibit the production of pro-inflammatory cytokines, regulate apoptosis, improve mitochondrial function, and activate endogenous antioxidant systems. Hops have a protective effect in cardiovascular diseases, metabolic disorders, neurodegenerative diseases and selected cancers through anti-inflammatory, anti-proliferative and metabolic mechanisms. In addition, hop compounds modulate the composition and activity of the gut microbiota, which promotes improved metabolic homeostasis. Despite relatively good intestinal absorption, systemic bioavailability remains limited; however, modern delivery systems significantly increase the stability and plasma concentrations of these compounds. **Conclusions:** Common hops have broad therapeutic potential due to their ability to regulate oxidative, inflammatory, metabolic and apoptotic processes at multiple levels. Their pleiotropic activity makes them a promising candidate for the prevention and treatment of chronic diseases. The development of delivery systems and consideration of the role of the gut microbiota may further increase its clinical application.

## 1. Introduction

Common hop (*Humulus lupulus* L.) is a perennial, dioecious climbing plant belonging to the family Cannabaceae [1,2,3]. It is characterized by a well-developed root system and long shoots that twine around supports in a clockwise direction, typically reaching heights of 10–18 m. Hop leaves are heart-shaped, arranged oppositely, and most often 3–5-lobed; the lobes have distinctly serrated margins, which constitutes one of the key morphological features of the species [2,3,4].

From the botanical perspective and with regard to the structure of the generative organs, a crucial characteristic of hop is its dioecy, meaning that male and female flowers are borne on separate plants. Female inflorescences form cone-like, spike-shaped structures commonly referred to as cones or strobiles, with a length of approximately 2.5–5 cm. Each cone consists of a central axis bearing bracts and bracteoles [1,2,4,5]. At the base of the bracteoles are characteristic lupulin glands (lupulin), which secrete a fine, yellow, resinous powder. These lupulin glands are the site of synthesis and accumulation of the key constituents responsible for the technological and biological value of hop raw material, primarily resins, essential oils, and a portion of flavonoids [1,2,3,4]. Hop is a wind-pollinated plant; however, in commercial cultivation, male plants are deliberately removed to prevent fertilization, as the presence of seeds is undesirable in the brewing process and leads to a reduction in the technological quality of the raw material [1,2,3,4,5].

The utility value of hop results from its complex chemical composition. A major fraction consists of resins (approximately 15–30%), which are divided into soft resins (alpha- and beta-acids) and hard resins [1,3,4]. During wort boiling, alpha acids (humulones) undergo isomerization to iso-alpha acids, which are directly responsible for the characteristic bitterness of beer [1,4,5,6]. Beta-acids (lupulones) do not isomerize in an analogous manner; however, they exhibit strong antibacterial properties, which is important both technologically and potentially functionally [1,2,3]. Another important group comprises essential oils (0.1–4.0%), including more than 1000 compounds, among which terpenes—such as myrcene, humulene, and caryophyllene—are particularly significant, as they shape the aroma and flavor of beer [1,2,3,5]. The chemical profile is further complemented by polyphenols (3–6%), including flavonoids and tannins, which contribute to beer stability and exhibit strong antioxidant activity. The most important prenylated flavonoid of hop is xanthohumol, frequently cited as one of the main compounds with bioactive potential [1,2,3,4,6,7].

The use of hop is multidirectional and has been known to humans since prehistoric times; nevertheless, brewing remains by far the dominant area of its application [2,4]. It is estimated that approximately 97–98% of global hop production is directed to the brewing industry, where hop constituents impart bitterness and aroma to beer, stabilize foam, and act as natural preservatives by inhibiting the growth of Gram-positive bacteria [1,2,3,4,5,6]. At the same time, hop holds an established position in traditional medicine and has also found applications in modern therapeutic approaches. It has long been used as a sedative and sleep-promoting agent, while contemporary studies indicate its potential relevance in the context of civilization-related diseases [3,4,5,6,8]. In particular, anti-inflammatory and antioxidant effects have been described, which may help mitigate the consequences of oxidative stress and inflammatory conditions [2,3,5,7,9]. Anticancer properties have also been reported, especially in relation to compounds such as xanthohumol, which exhibits the ability to inhibit the proliferation of cancer cells [2,3,4,7]. Attention has likewise been drawn to the possible role of iso-alpha acids in improving lipid and glucose metabolism, which is relevant in the context of metabolic syndrome, obesity, and type 2 diabetes [2,3,6]. In addition, in menopausal phytotherapy, particular importance is attributed to 8-prenylnaringenin, considered one of the most potent known phytoestrogens, which is associated with the potential alleviation of menopausal symptoms [1,2,3,4,6]. Beyond brewing and medicinal applications, hop extracts are also used in cosmetics (e.g., perfumes and creams) and as natural insecticides [1,2,3,4]. It is worth emphasizing that young hop shoots are sometimes consumed as a vegetable, representing a less common but historically and culturally significant direction of use. By-products associated with hop processing, such as spent hops, may be utilized as fertilizer or as an animal feed additive, which aligns with approaches aimed at valorizing waste streams and using biomass in a more sustainable manner [2].

**Figure 1:**
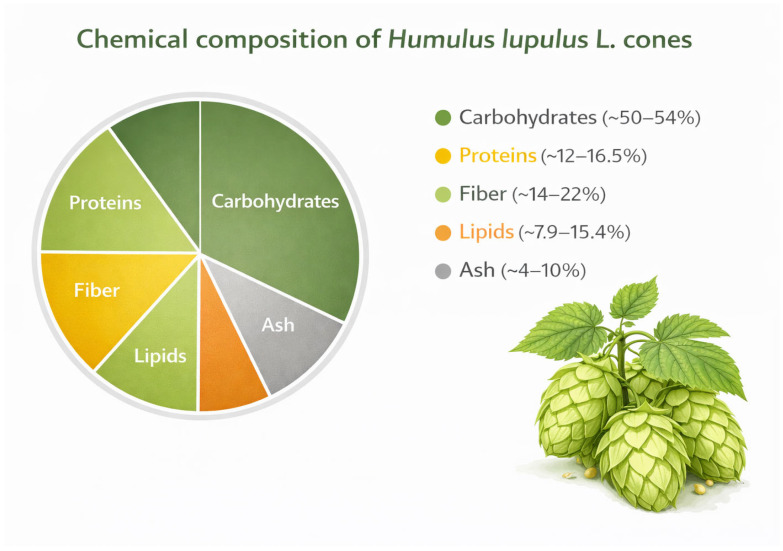
Chemical composition of *Humulus lupulus* L. cones.

The cultivation requirements of hop are closely related to climatic conditions, particularly day length, summer temperatures, and the total amount and distribution of precipitation. For this reason, hop is cultivated mainly in temperate climate zones, within the belt between 35° and 55° latitude in both the Northern and Southern Hemispheres [1,3,5]. On the global market, Germany and the United States dominate, together accounting for approximately 75–80% of total hop production. Other important hop-growing countries include China, the Czech Republic, and Poland, which also contribute to the supply of raw material and, in practice, constitute important links in the global hop production chain [1,3,4] (Figure 1).

## 2. Materials and Methods

The literature included in this narrative review was identified through a structured search of the PubMed/MEDLINE, Scopus, Embase, and Google Scholar databases. The search strategy encompassed original research articles and review papers published between 2000 and 2026, with the final search conducted in January 2026 and particular emphasis on studies from the last decade (2015–2026). Combinations of keywords were applied using Boolean operators (“AND”, “OR”), including but not limited to: *Humulus lupulus*, hops, xanthohumol, prenylated flavonoids, bitter acids, polyphenols, oxidative stress, inflammation, NF-κB, Nrf2, AMPK, PPAR, gut microbiota, cardioprotective, neuroprotective, metabolic, estrogen-like, anticancer, and immunomodulatory effects. Additional relevant publications were identified through manual screening of reference lists from selected articles.

Studies were included if they directly investigated hops or hop-derived bioactive compounds and provided relevant information on their biological activity, molecular mechanisms, bioavailability, metabolism, or potential health effects. Eligible publications comprised in vitro, in vivo, and human studies, written in English and available as full-text articles. Publications were excluded if they demonstrated limited methodological quality (e.g., lack of control groups, insufficient experimental design, or unclear outcome reporting), focused on unrelated polyphenols, or were restricted to editorials, conference abstracts, commentaries, or non–peer-reviewed sources. Duplicate records were removed during the selection process.

All included articles were independently assessed for scientific relevance and consistency with the aims of this review. The selection process involved an initial screening of titles and abstracts, followed by full-text evaluation of potentially eligible studies. Any discrepancies in study selection were resolved through discussion between the authors. Extracted data covered modulated signaling pathways, biological outcomes, dose–response relationships, metabolic interactions, and the influence of hop-derived compounds on inflammatory, oxidative, and hormonal processes. As this work represents a narrative review, no formal risk-of-bias assessment or statistical meta-analysis was performed. However, the review process was conducted in accordance with general principles of structured literature reviews, with elements inspired by PRISMA guidelines to enhance transparency and reproducibility. Ethical approval and study registration were not required.

## 3. Chemical Composition

Common hop (*Humulus lupulus* L.) is characterized by a complex chemical profile that includes both compounds of nutritional character (primary metabolites) and numerous secondary metabolites responsible for its sensory and biological properties. The key determinants of hop “strength” and character are the lupulin glands, in which resins (bitter acids), essential oils, and polyphenols are primarily accumulated. This composition is variable and depends on the cultivar, cultivation conditions, harvest time, technological processing, and storage, making hops a raw material highly sensitive to external factors [10,11,12].

Among the primary metabolites, structural and building components predominate. Carbohydrates constitute the largest proportion of dry matter (approximately 51–54%), including glucose, fructose, maltose, and raffinose [10,13]. Another important fraction is proteins, accounting for approximately 12–16.5% of dry matter, which are relevant to nutritional value and also indirectly influence technological processes [10,13]. Hop cones also contain lipids (7.9–15.4%, with higher values reported for some cultivars) [13,14] and crude fiber (14.6–22.2%), which contributes to the structural integrity of the raw material [13]. Ash content ranges from 4.4 to 10.3%, reflecting the contribution of mineral components [13,14]. Epicuticular waxes are also present on the surface of leaves and other plant structures; these are composed mainly of primary alcohols (54%), triterpenoids (22%), and fatty acids (13%) and serve a protective function [15].

From the perspective of biological activity and technological relevance, secondary metabolites are of greatest importance and are particularly concentrated in lupulin. This group includes hop resins (10–30% of dry matter), which are divided into soft and hard fractions [10,14]. The most valuable compounds are found in the soft fraction and include alpha acids (humulones, approximately 3–15%) and beta acids (lupulones, approximately 3–10%), which are directly responsible for hop bitterness and antimicrobial activity [10,11]. The hard fraction accounts for approximately 1.5–2.5% of dry matter and represents a more stable portion of the resins, although it is of lesser importance for the fresh aromatic and sensory characteristics of hops [14].

Another key fraction consists of essential oils, which make up approximately 0.1–4.0% of dry matter and contain a very large number of volatile compounds (over 1000 identified compounds) [10,12,16]. The oil fraction is dominated by terpene hydrocarbons (50–80%), including monoterpenes (myrcene, pinene) and sesquiterpenes (α-humulene, β-caryophyllene) [14,17]. The remaining compounds include oxygenated constituents such as alcohols (linalool and geraniol), aldehydes, ketones, esters, and small amounts of sulfur-containing compounds. This group is directly responsible for hop aroma, fragrance profile, and some relaxing and antibacterial effects [12,17].

Polyphenols (3–6% of dry matter) also represent an important component of hops and include both compounds with high antioxidant activity and hop-specific prenylated compounds [14,17]. Of particular importance are prenylated flavonoids, with xanthohumol (0.1–1.7% of dry matter) being the most characteristic, along with isoxanthohumol and 8-prenylnaringenin, one of the most potent known phytoestrogens [11,14]. In addition, hops contain flavonols (e.g., quercetin, kaempferol, and myricetin, often in glycosylated forms) [11,17], phenolic acids (including caffeic, chlorogenic, and ferulic acids) [14,17], and tannins (proanthocyanidins). Polyphenols largely determine the antioxidant potential of hops and contribute to their anti-inflammatory effects [10,17].

The biological effects of hops are closely linked to their chemical composition, as individual fractions are responsible for distinct mechanisms of action. Bitter acids exhibit particularly strong antibacterial properties, especially against Gram-positive bacteria, and may also support anti-inflammatory and sedative effects [10,13,17,18]. Xanthohumol and other prenylflavonoids are responsible for antioxidant activity and potential anticancer mechanisms, including effects on cell survival, oxidative stress, and inflammatory signaling pathways [10,17,18]. In contrast, oil-derived terpenes shape relaxing effects, aroma, and antimicrobial and antifungal properties [10,12,17,19], while flavonols and phenolic acids support broadly defined protective effects, including potential cardioprotective and neuroprotective actions [14,20].

The content of individual hop constituents is primarily determined by genetic factors, namely the cultivar [10,12,16,21]. The genotype determines whether a given variety is aromatic, characterized by lower alpha acid content and a richer oil profile, or bitter, with a high alpha acid content often exceeding 8–15%. Varieties also differ in terpene proportions, the presence of specific chemical markers (e.g., selected sesquiterpenes), and their capacity to accumulate xanthohumol. In practice, this means that two cultivars grown even under similar environmental conditions may exhibit completely different functional profiles and biological potential [10,17,21].

Another major factor influencing composition is terroir and the cultivation environment, including geographic location, soil type, mineral availability, and climatic conditions [10,11,13,19,20]. Temperature, precipitation, and sunlight modulate the synthesis of bitter acids and polyphenols, while seasonal variability in weather conditions can result in noticeable compositional fluctuations even within a single plantation [11,16,20,22]. In addition, climate change, such as increasing temperatures, may negatively affect alpha acid accumulation, directly influencing the quality and commercial parameters of the raw material [11,13,20,22].

The degree of cone maturity and the timing of harvest are also critical, as the profile of hop compounds changes during development. Individual terpene groups may appear at different stages, and the final essential oil content largely depends on whether harvesting occurs within the optimal window [12,16]. In practice, harvest timing affects not only aroma intensity but also the proportions of volatile compounds, thereby determining raw material quality and suitability for industrial applications [14,16,20].

After harvest, the chemical composition of hops is strongly shaped by technological processing and the physical form of the product. Drying is a critical step, as excessively high temperatures lead to losses of essential oils and accelerate the degradation of sensitive fractions [16]. Pelletization increases material fragmentation and may cause rupture of lupulin glands, facilitating the release of compounds but simultaneously increasing their exposure to oxygen and accelerating oxidative reactions [12,13]. The extraction method is also important, as the choice of solvent and processing parameters selectively isolates specific fractions (resins, oils, polyphenols), thereby altering both their concentrations and relative proportions [11,12,13,14,16,20].

The most destructive and often underestimated factor is storage, which determines the stability of the hop chemical profile over time. Temperature, oxygen availability, and storage duration lead to the gradual degradation of alpha acids and changes in the oil profile. Particularly sensitive monoterpenes (e.g., myrcene) may rapidly decline, while oxidative processes result in an increased proportion of oxygenated compounds (e.g., alcohols and epoxides), completely altering the aroma of the raw material. As a result, improperly stored hops may lose their original character even when they formally still meet basic quality criteria [12,16].

Chemical composition is also influenced by biotic factors such as diseases and pests, which induce physiological stress in the plant. Defensive responses may lead to changes in the production of secondary metabolites, particularly volatile and protective fractions, and may also affect surface structures such as the composition of epicuticular waxes. This means that pathogen or pest pressure may not only reduce yield but also modify the chemical quality of the raw material and its technological and sensory value [15,16].

## 4. Bioavailability and Limitations of Biological Activity

The bioavailability of bioactive compounds present in hops, particularly prenylflavonoids such as xanthohumol (XN) and 8-prenylnaringenin (8-PN), is considered to be significantly limited following oral administration. Despite the high biological activity observed in in vitro and preclinical studies, the concentrations of these molecules achieved in systemic circulation are often low, which represents one of the key challenges in assessing their actual health-promoting potential in humans. This discrepancy between experimental activity and systemic exposure represents a major limitation in translating preclinical findings into clinically relevant effects, as the concentrations required to achieve biological activity in vitro are often substantially higher than those observed in vivo [23,24,25].

One of the main factors limiting the bioavailability of prenylflavonoids is their relatively low water solubility. These compounds exhibit a pronounced hydrophobic character, which reduces their ability to dissolve in the aqueous environment of the gastrointestinal tract and limits their passage across the intestinal barrier via passive diffusion. In practice, this means that even with high dietary intake, the fraction available for absorption remains small, and the absorption process is highly sensitive to digestive conditions and the composition of intestinal contents. Consequently, the effective systemic dose reaching circulation may represent only a small proportion of the administered amount, which further complicates the interpretation of dose–effect relationships in human studies [26,27].

Another important limitation is associated with the cellular barriers of the intestinal epithelium. Xanthohumol shows a strong affinity for binding to cytosolic proteins of enterocytes and may also incorporate into the lipid bilayer of cell membranes, which hinders its rapid transmembrane transport. As a result, a portion of the molecules is “retained” at the level of enterocytes before reaching the portal circulation. These limitations are physicochemical and structural in nature, indicating that the mere presence of a compound in plant material does not determine its biological availability. This phenomenon may additionally contribute to local intestinal effects that are not necessarily reflected by plasma concentrations, which should be considered when interpreting systemic versus gut-mediated mechanisms of action [23,25,28].

Bioavailability is also influenced by subtle structural differences between compounds with very similar chemical structures. For example, 8-PN may exhibit noticeably higher bioavailability than its isomer 6-PN, despite their close chemical relationship [23,27]. A similar phenomenon is observed for hop resins, as β-acids tend to be absorbed less efficiently than α-acids, which may result from their greater lipophilicity and more intensive metabolism. These relationships emphasize that the bioavailability of hop-derived compounds is strongly determined by both molecular properties and metabolic fate in the body. Such differences may partly explain variability in biological responses reported across studies, particularly when different hop fractions or preparations are used [23,25].

After absorption, hop bioactive compounds undergo intensive biotransformation, which markedly reduces the concentration of their free forms in plasma. Metabolism includes phase I processes (e.g., oxidation) and phase II reactions, dominated by glucuronidation and sulfation [23,29,30]. Consequently, prenylflavonoids circulate in the bloodstream almost exclusively as conjugates (glucuronides and sulfates), while unconjugated forms are usually difficult to detect. This has important interpretative implications, as the biological activity of free forms may differ from that of metabolites, and physiological effects depend on the chemical forms actually present in vivo. Therefore, it cannot be assumed that biological effects observed for native compounds directly correspond to those exerted by their circulating conjugated forms [31,32].

A characteristic feature of the pharmacokinetics of many hop-derived compounds is enterohepatic recirculation, suggested by a biphasic plasma concentration profile (the occurrence of two peaks). This mechanism results from the secretion of metabolites into bile, their passage into the intestine, and subsequent reabsorption, which may prolong systemic exposure to a given compound [23,25,29,32]. At the same time, the dominant routes of elimination are fecal and biliary excretion, whereas urinary excretion usually plays a minor role [23,29,33,34]. A relatively long half-life, often exceeding 20 h, may favor the persistence of metabolites in the body during regular intake, potentially increasing the likelihood of biological effects despite limited absorption of a single dose. However, prolonged circulation of metabolites does not necessarily correspond to high pharmacological activity, particularly if the active forms are present in low concentrations [29,35,36].

The bioavailability and biological effects of hop compounds also depend on the form of administration and the food matrix in which they are delivered. It has been shown that incorporation of xanthohumol into a protein-rich matrix (e.g., rice protein) may increase its plasma concentration compared with conventional hop powder, highlighting the importance of interactions between plant compounds and food components [29]. Importantly, bioavailability may also differ depending on whether a compound is administered as a pure substance or as part of a complex extract. In preclinical studies, pure xanthohumol exhibited higher bioavailability than an equivalent dose administered within a prenylflavonoid extract, suggesting that other components of the mixture may limit absorption through competition, binding, or modulation of solubility. These observations indicate that formulation strategies and delivery systems play a critical role in determining actual systemic exposure and should be considered when comparing experimental and clinical outcomes [29,37].

An increasingly emphasized factor is the role of the gut microbiota in the activation of selected hop metabolites. Intestinal bacteria can convert certain compounds, such as isoxanthohumol, into the strongly estrogenic 8-PN, meaning that the final magnitude of the biological effect depends not only on the dose but also on the individual composition of the host microbiome. This phenomenon explains the considerable interindividual variability in response and supports the need to consider microbiological aspects in the design of intervention studies and in the interpretation of results. This variability may contribute to inconsistent results observed in human studies and highlights the importance of personalized responses to hop-derived compounds [24,38,39].

**Figure 2:**
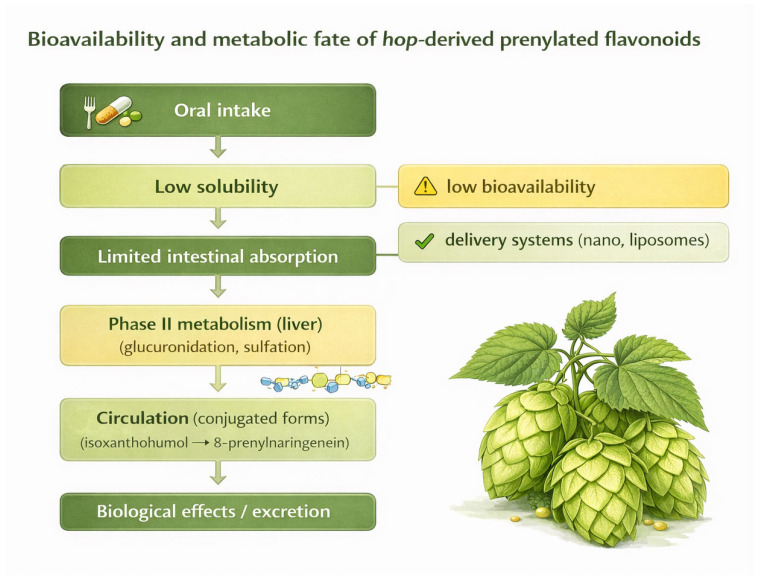
Bioavailability and metabolic fate of hop-derived prenylated flavonoids.

Due to these limitations, strategies to improve the bioavailability of hop-derived compounds through modern delivery systems are being intensively developed. Promising approaches include micellar solubilization, which enhances the solubility and transport of hydrophobic molecules across the intestinal barrier, and the formation of cyclodextrin complexes that improve stability and dispersion in aqueous environments [23,25,26,29]. Increasing attention is also being given to nanotechnology-based methods, such as liposomes, nanoemulsions, and nanoparticles, which can protect compounds from degradation in the gastrointestinal tract and prolong their circulation time. In addition, the use of so-called absorption enhancers (e.g., piperine) may reduce metabolic enzyme activity and increase intestinal availability; however, these strategies require careful evaluation of safety and potential interactions. Nevertheless, despite these promising approaches, their clinical relevance and long-term safety require further validation in human studies [25,26] (Figure 2).

## 5. Molecular Mechanisms of Action of Hop-Derived Compounds

### 5.1. Antioxidant Activity

Common hop constitutes an exceptionally valuable source of natural compounds with antioxidant properties that may play an important role in reducing oxidative stress at the cellular level. Its antioxidant activity is associated with the high content of secondary metabolites, such as polyphenols, bitter acids, and prenylated flavonoids, which act both through direct neutralization of free radicals and by inhibiting processes leading to their formation. As a result, hops are perceived not only as a bittering and aromatic raw material, but also as a component supporting health, food product stability, and protection of cells against oxidative damage [13,40,41,42,43,44,45].

One of the most important hop-derived compounds with high antioxidant potential is xanthohumol (XN/XH), a prenylated flavonoid that represents the dominant member of this group in hop cones [46,47]. This compound is a very potent antioxidant, effectively neutralizing free radicals and limiting the initiation and propagation of chain reactions associated with lipid oxidation. Studies have demonstrated that xanthohumol exhibits 8.9-fold stronger activity than Trolox in scavenging hydroxyl radicals and 2.9-fold stronger activity in neutralizing peroxyl radicals [40,42,46]. In practice, this indicates that xanthohumol may exert beneficial effects in terms of protecting cell membranes and lipid stability, including limiting the oxidation of lipoprotein fractions such as LDL, which is relevant in the prevention of processes associated with atherosclerosis and chronic inflammation. It is worth emphasizing that xanthohumol is often described as a compound with a broad spectrum of activity, encompassing anti-inflammatory effects and modulation of cellular pathways related to stress responses [13,42,47,48].

Another important component of the antioxidant profile of hops consists of bitter acids (alpha acids and beta acids). Despite their widespread use in the brewing industry due to their bittering and sensory properties, they exhibit high biological activity. They participate in free radical scavenging and limit lipid peroxidation. In food systems, the inhibition of peroxidation slows rancidity, whereas in the context of human health, it may exert a strong protective effect on lipid-rich structures, such as cell membranes and lipoproteins. In some studies, humulone (alpha acids) demonstrated significant antioxidant potential, sometimes comparable to or discussed alongside classical plant-derived antioxidants, which highlights the uniqueness of this hop fraction relative to other raw materials [40,49,50,51,52].

Another significant group comprises phenolic compounds present in hops, including flavonols (e.g., quercetin and kaempferol), flavan-3-ols (e.g., catechin and epicatechin), and phenolic acids (such as ferulic, caffeic, and chlorogenic acids) [43,44,49,51]. Their antioxidant activity depends on several complementary mechanisms. The first involves scavenging free radicals through electron or hydrogen atom donation; the second involves an enhanced ability to chelate transition metal ions (e.g., Fe^2+^ and Cu^2+^), which, in the presence of peroxides, intensify hydroxyl radical formation and can catalyze oxidation reactions. The third mechanism involves the inhibition of pro-oxidative enzymes (e.g., lipoxygenases), thereby reducing the rate of generation of oxidation products in food matrices. For this reason, the abundance of phenolic compounds in hops is considered an important pillar of their protective activity [42,43,51,53].

In practice, the antioxidant properties of hops and their extracts are evaluated using a range of analytical methods, such as DPPH, ABTS, FRAP, CUPRAC, and ORAC. These assays differ in their reaction mechanisms and the type of “model” oxidant used; therefore, the parallel application of several methods is often recommended to obtain a more comprehensive picture of antioxidant activity. Most of these assays are based on two main mechanisms: hydrogen atom transfer (HAT) or single-electron transfer (SET) [42,51,52,54]. Importantly, the final outcome strongly depends on the chemical composition of the extract and on whether its constituents act additively, synergistically, or antagonistically. Synergism is particularly relevant in the case of hops, as a mixture of prenylflavonoids, bitter acids, and simple phenolics may produce an effect greater than the sum of the activities of individual components assessed separately [54].

However, the antioxidant activity of hops is not constant and depends on many biological and technological factors. One of the key determinants is genotype (cultivar), as differences in polyphenol profiles and in the proportions of α- and β-acids cause individual cultivars to vary significantly in reducing potential and the ability to neutralize reactive oxygen species. The literature often emphasizes that selected aromatic cultivars may exhibit particularly high antioxidant activity, which is relevant both for food applications and for the development of functional extracts. Another important aspect is the plant part, as the highest concentrations of active compounds are usually found in cones (especially in lupulin), although leaves (particularly young ones), seeds, and roots may also represent valuable alternative sources of phenolics and other bioactive substances [51,54,55].

Extraction conditions also play a major role in shaping antioxidant activity, including the choice of solvent, its concentration, temperature, time, and even pH. The use of alcohol–water mixtures (e.g., ethanol) generally enables more efficient isolation of phenolic compounds with diverse polarity, whereas water alone (especially in the case of infusions) may yield a different spectrum of compounds depending on process parameters. The influence of pH is particularly important, as some phenolics (e.g., catechins) may exhibit variable stability in acidic or more alkaline environments, which directly affects the actual amount of active substances available in the extract. Consequently, hop extracts obtained using different methods may have similar dry matter content but markedly different antioxidant activity in analytical assays [13,56,57].

From an industrial perspective, it is also important how hops respond to technological processing. Drying of cones may lead to some losses of sensitive compounds; however, in many cases, these changes do not necessarily result in a significant reduction in overall antioxidant activity, particularly when key fractions remain stable. Similarly, processes such as pelletization are generally not considered destructive to an extent that would completely eliminate the antioxidant potential of the raw material, although the final effect always depends on specific conditions, including temperature, oxygen exposure, and contact time. This is relevant from a technological standpoint, as it allows hops to be used in forms that are logistically convenient and storage-stable without loss of their most important biological properties [13,55].

The wide range of potential applications of hops results from the fact that their antioxidants reduce oxidative stress in various model systems, which translates into applications in food technology. Hop extracts may serve as natural additives that improve fat stability and inhibit lipid oxidation, a process underlying rancidity and sensory deterioration in many products. Consequently, hops are used, for example, in meat products, bakery goods, or products with increased fat content, where the aim is to slow oxidative degradation, maintain color, and limit the formation of undesirable aromas. This approach is consistent with the current trend of replacing synthetic antioxidants with plant-derived alternatives [40,43].

In the context of prevention and potential support of human health, hops have attracted considerable interest due to their ability to enhance endogenous antioxidant defense mechanisms. Oxidative stress is recognized as one of the factors accompanying the development of many chronic diseases, including cardiovascular disorders, metabolic disturbances, and neurodegenerative processes. Compounds such as xanthohumol may influence signaling pathways involved in cellular responses to oxidative damage, which may potentially translate into protection of neurons and vascular structures. However, it should be emphasized that biological efficacy in the human body depends not only on the strength of activity observed under laboratory conditions, but also on bioavailability, metabolism, dose, and duration of exposure; therefore, interpretation of health effects requires caution and consideration of study quality [28,40,51,53].

### 5.2. Anti-Inflammatory Activity

Common hop, and specifically the compounds it contains, such as bitter acids, prenylated flavonoids, and essential oils, exhibit a range of strong effects in counteracting inflammatory processes in the human body. Anti-inflammatory effects are mediated through the multidirectional modulation of signaling pathways that regulate inflammatory responses at the cellular and molecular levels [58,59]. In particular, these compounds act on the NF-κB, Nrf2, and MAPK pathways and regulate the expression of pro-inflammatory enzymes such as COX-2 and iNOS. Through interactions with the above-mentioned mechanisms, hops demonstrate high protective and regulatory potential toward tissues and organs that are particularly exposed to chronic inflammation, including the gastrointestinal tract, joints, skin, and the central nervous system [40,50,60].

The primary and key biologically active fraction consists of bitter acids, including alpha acids, beta acids, and their isomers, particularly iso-alpha acids [40,59]. These substances exhibit strong anti-inflammatory activity by inhibiting cyclooxygenase-2 activity and limiting the induction of pro-inflammatory genes regulated via the NF-κB pathway. Inhibition of IKK kinase phosphorylation prevents the translocation of NF-κB to the cell nucleus, resulting in reduced expression of pro-inflammatory cytokines such as TNF-α and IL-6. This leads to decreased synthesis of prostaglandins, including PGE2, and attenuation of inflammatory symptoms such as edema, pain, and tissue damage [40,61,62].

An important group of chemically active compounds is represented by prenylated flavonoids, among which xanthohumol is considered to have the greatest biological significance. This compound exhibits the ability to inhibit macrophage activation, reduce the synthesis of pro-inflammatory cytokines, and limit angiogenic processes accompanying chronic inflammation [58,63]. In addition, xanthohumol activates the Nrf2–ARE pathway, leading to the induction of phase II detoxifying enzymes such as HO-1 and NQO1, thereby increasing cellular resistance to oxidative stress, which is one of the main factors initiating and sustaining inflammation [60].

Essential oils are a source of terpenes with documented anti-inflammatory and analgesic activity. β-Caryophyllene acts as an agonist of CB2 receptors, resulting in immunomodulation and inhibition of the release of key inflammatory mediators (TNF-α, IL-1β, IL-6), and attenuation of pain signaling. Its activity is not associated with psychoactive effects, which increases its safety of use. Myrcene exhibits analgesic effects through modulation of neural conduction and inhibition of prostaglandin and nitric oxide synthesis, thereby reducing the sensitivity of nociceptive receptors. Acting in parallel, both compounds contribute to comprehensive suppression of inflammatory responses and pain perception [58,64,65].

Within the gastrointestinal tract, hop extracts exhibit protective effects on the gastric and intestinal epithelium. In vitro studies have shown that hydroalcoholic hop extracts inhibit interleukin-8 secretion in gastric epithelial cells, thereby limiting neutrophil recruitment and the development of chronic inflammation [66]. Prenylated flavonoids have been shown to preserve the integrity of the intestinal barrier, preventing damage induced by pro-inflammatory cytokines such as TNF-α and IL-1β. It has also been confirmed that in animal models of colitis, xanthohumol reduces COX-2 expression and the levels of inflammatory cytokines, which translates into alleviation of clinical symptoms of the disease [40,67].

The anti-inflammatory properties of hops are also of significant importance in the context of degenerative joint diseases. Studies conducted on human chondrocytes have demonstrated that hop extracts inhibit the expression of genes encoding enzymes involved in cartilage degradation, including matrix metalloproteinases. At the same time, a reduction in nitric oxide and prostaglandin production has been observed, indicating a potential role of hops in pain relief and slowing the progression of osteoarthritis. Clinical efficacy has been confirmed in studies involving patients, in whom preparations based on reduced iso-alpha acids led to a decrease in pain symptoms [40,61,68].

Increasing attention is also being paid to the influence of hop compounds on neuroinflammation and the functioning of the central nervous system. Iso-alpha acids have been shown to modulate microglial activity, promoting an anti-inflammatory phenotype and enhancing phagocytic processes. In animal models of neurodegenerative diseases, a reduction in beta-amyloid deposition and decreased levels of pro-inflammatory cytokines in brain regions responsible for memory and cognitive functions have been observed, suggesting a potential neuroprotective effect [63].

In dermatology, hop extracts are used due to their anti-inflammatory, antibacterial, and photoprotective properties. Compounds such as humulones and lupulones exhibit activity against bacteria responsible for acne and skin infections, while simultaneously limiting inflammatory responses induced by UV radiation. Studies in keratinocytes confirm the ability of hop extracts to inhibit interleukin-6 production, which further supports their potential in skin care and protection [58,59,69].

A significant challenge in the application of hop compounds remains their limited bioavailability. In response to this issue, modern encapsulation strategies such as nanoliposomes and polysaccharide microcapsules are being developed to protect bioactive compounds from degradation in the gastrointestinal tract and to increase their biological availability. Studies indicate that such technological solutions significantly enhance the anti-inflammatory activity of hop flavonoids, particularly within the intestines and joints, opening new perspectives for their application in functional foods and nutraceuticals [40,68,70,71].

### 5.3. Modulation of Metabolic and Cellular Processes

Modulation of metabolic and cellular processes by phytochemicals, particularly those derived from hops, occurs through complex and multidirectional molecular mechanisms involving the regulation of nuclear receptor activity, key signaling pathways, and gene expression responsible for energy homeostasis and cell proliferation. Compounds such as isohumulones, xanthohumol, and their derivatives exhibit pleiotropic effects, simultaneously influencing glucose and lipid metabolism, inflammatory processes, mitochondrial function, and the cell cycle, which makes them important modulators of organismal physiology [72,73].

One of the key areas of activity of hop-derived compounds is their influence on insulin resistance and carbohydrate–lipid metabolism. Isohumulones and their reduced derivatives demonstrate the ability to activate PPARα receptors and partially activate PPARγ, resulting in improved tissue insulin sensitivity and reduced markers of insulin resistance [73,74,75]. These effects lead to normalization of fasting insulin levels, significant improvement in glucose tolerance, and attenuation of metabolic disturbances induced by a high-fat diet [72,73,74]. Xanthohumol additionally inhibits the activity of digestive enzymes responsible for carbohydrate breakdown, which contributes to stabilization of postprandial glycemia. In parallel, a beneficial effect on lipid metabolism is observed, manifested by reduced concentrations of triglycerides, free fatty acids, and LDL cholesterol, along with an increase in HDL cholesterol, which partly results from inhibition of de novo lipogenesis pathways in the liver [72,73,76,77].

Such targeted modulation of metabolic processes is of significant importance in the prevention of civilization-related diseases, particularly metabolic syndrome, obesity, and type 2 diabetes. Hop-derived compounds limit body weight gain and visceral fat accumulation, among others, by stimulating adiponectin secretion and activating AMP-activated protein kinase (AMPK), which promotes energy expenditure and fatty acid oxidation [72,74,77,78]. Improvement in the lipid profile, combined with concurrent anti-inflammatory effects, as reflected by reduced expansion of pro-inflammatory cytokines, contributes to the inhibition of atherosclerosis development and improvement in endothelial function. As a result, normal nitric oxide bioavailability is restored, and vascular elasticity is improved, which is relevant for cardioprotection [72,79,80]. Over the long term, such effects may slow the progression of the prediabetic state and reduce the risk of developing overt diabetes [72,80].

Another important aspect of the activity of hop-derived compounds is their effect on cell proliferation and apoptosis. Xanthohumol exhibits the ability to inhibit and promote differentiation of preadipocytes and to induce programmed cell death in mature adipocytes, which represents one of the mechanisms limiting excessive accumulation of adipose tissue. This process is associated with modulation of cellular redox balance, increased production of reactive oxygen species, and activation of the caspase cascade [74,77]. Similar mechanisms are observed in cancer cells, where hop phytochemicals reduce proliferation and survival of various tumor cell types by inducing apoptosis via both mitochondrial pathways and activation of death receptors [65].

Regulation of the cell cycle constitutes another element of the activity of the compounds discussed. Decreased expression of proliferation markers and effects on proteins controlling successive phases of the cell cycle lead to inhibition of cell division [65,77]. In addition, some hop flavonoids exhibit epigenetic activity through modulation of DNA methylation and histone acetylation, resulting in persistent changes in the expression of genes involved in the control of cell growth and differentiation [65,72].

At the level of cellular signaling, particular importance is attributed to the modulation of axes regulating lipid and glucose metabolism. Hop-derived compounds influence the activity of nuclear receptors and proteins involved in lipolysis and lipid uptake from the circulation, thereby contributing to reduced triglyceride concentrations [81]. Activation of the AMPK and PPARα pathways promotes fatty acid oxidation in the liver while simultaneously inhibiting lipogenic processes [82]. In addition, some xanthohumol derivatives exhibit the ability to induce mild mitochondrial uncoupling, leading to increased cellular respiration rate and energy expenditure, which represents a mechanism counteracting obesity [79].

### 5.4. Hormonal and Estrogen-like Activity

The hormonal activity of common hop and its derivatives results primarily from the presence of 8-prenylnaringenin (8-PN), a prenylated flavanone that is currently regarded as the most potent plant-derived phytoestrogen [83,84,85]. This compound occurs naturally in the lupulin glands of female hop cones; however, in plant raw material, it is usually present in very small amounts, often below 0.01% of dry matter [35,85,86]. Despite its low concentration in the starting material, its actual biological activity in the human body may be significant, owing to chemical and metabolic processes occurring both during hop processing and within the gastrointestinal tract and liver. The estrogenic potency of 8-PN is estimated to be approximately 50 times higher than that of classical phytoestrogens such as genistein or coumestrol, which makes it a compound of exceptional biological and clinical relevance [83,84,86].

The hormonal effects of hops are based on a complex scheme of formation and biotransformation of prenylated flavonoids. One of the key stages is thermal isomerization, which occurs during technological processes such as wort boiling. During this process, the estrogenically inactive desmethylxanthohumol (DMX) undergoes spontaneous conversion into a mixture of 6-prenylnaringenin (6-PN) and the biologically active 8-prenylnaringenin [39,83,84]. At the same time, the most abundant hop flavonoid, xanthohumol (XN), undergoes cyclization to isoxanthohumol (IX) in the acidic environment of the stomach. The gut microbiota plays a particularly important role in the estrogen-like activity of hops [35,39,83,84]. In the large intestine, isoxanthohumol may undergo O-demethylation to 8-PN with the participation of specialized bacteria, especially Eubacterium limosum [39,83,84,87]. The efficiency of this process is highly individual and depends on the composition of the intestinal microbiota, leading to the identification of populations with high, moderate, or low capacity for endogenous 8-PN production [39,83,85,86]. In addition, demethylation of isoxanthohumol to 8-PN may also occur in the liver, where the cytochrome P450 enzyme CYP1A2 is involved. The complexity of these transformations means that the hormonal effect of hops is determined by both the dose of the raw material and the metabolic characteristics of the individual [83,85].

At the cellular level, 8-prenylnaringenin acts by mimicking natural estrogens, particularly 17β-estradiol. This compound binds to estrogen receptors, showing a markedly stronger affinity for the ERα subtype than for ERβ. The preference for ERα is biologically significant, as this receptor predominates in tissues such as the uterus, mammary gland, and bone [83,84,85,86,88]. The affinity of 8-PN for ERα is approximately 100 times higher than that of genistein, which further highlights its exceptional estrogenic potency [39,85]. For this reason, 8-PN is classified as a natural selective estrogen receptor modulator (SERM), capable of exerting agonistic activity in some tissues and potentially antagonistic effects in others [83,84,85]. In addition to classical genomic mechanisms, 8-PN may also act via non-genomic pathways, including inhibition of aromatase activity, the enzyme responsible for the conversion of androgens to estrogens [35,83,84].

Results from clinical and in vivo studies confirm the significant role of the estrogen-like activity of hops in alleviating menopausal symptoms. Placebo-controlled studies have shown that administration of hop extract standardized to 100 µg of 8-PN per day leads to a marked reduction in hot flashes, night sweats, anxiety, and sleep disturbances after 6–12 weeks of supplementation [35,84,86,89]. One study conducted in a group of 120 women demonstrated nearly a 90% reduction in early vasomotor symptoms after 90 days of hop extract use, indicating high efficacy of this compound in the context of climacteric symptoms [35,84].

The estrogen-like effects of 8-PN are also reflected in its influence on bone tissue metabolism. In vitro studies have shown that 8-prenylnaringenin stimulates alkaline phosphatase activity in osteoblasts, thereby promoting bone-forming processes, while simultaneously inhibiting the differentiation and activity of osteoclasts responsible for bone resorption [83,84,86,90]. In a 48-week clinical study in women with osteopenia, supplementation with hop extract containing 100 µg of 8-PN, administered in combination with calcium and vitamin D, resulted in an approximately 1% increase in total body bone mineral density (BMD) compared with the placebo group, confirming the potential relevance of hops in osteoporosis prevention [87].

The hormonal activity of hops and their derivatives is also associated with observed anticancer activity. Both xanthohumol and 8-PN exhibit the ability to inhibit the proliferation of cancer cells of the breast, prostate, colon, and cervix, mainly through induction of apoptosis and activation of endoplasmic reticulum stress [39,85,86,91]. Importantly, xanthohumol may act antagonistically against the estrogenic effects of mycotoxins such as zearalenone, which may occur as food contaminants and exhibit their own estrogen-like activity [39,85].

Despite the documented biological benefits, the high estrogenic activity of 8-PN also raises potential health risks. Administration of very high doses of 8-PN may lead to stimulation of the endometrium, resulting in endometrial hyperplasia, which in some cases has manifested as postmenopausal bleeding in women using hop supplements with imprecisely defined composition [35,89,90]. Moreover, hop derivatives may affect the activity of cytochrome P450 enzymes, including CYP1A2 and CYP2C9, creating a risk of interactions with drugs such as warfarin or caffeine [84,85]. In addition, in vitro studies suggest that high concentrations of 8-prenylnaringenin may impair oocyte maturation, indicating the need for caution among women of reproductive age, particularly those using hop supplements marketed as agents influencing hormonal balance or breast development [35,39,84,89].

From a safety perspective, the estrogenic activity of 8-prenylnaringenin requires careful consideration, particularly in the context of long-term supplementation. Although clinical studies have demonstrated beneficial effects in alleviating menopausal symptoms, the available data are still limited in duration and scope, and do not fully address long-term hormonal safety. Prolonged exposure to compounds with high affinity for estrogen receptors, especially ERα, may potentially influence hormone-dependent tissues, including the endometrium and breast, which necessitates cautious interpretation of results [35,39,83,84,86,90].

Furthermore, the potential for interactions with pharmacotherapy should be taken into account. Hop-derived compounds may modulate the activity of cytochrome P450 enzymes, including CYP1A2 and CYP2C9, which are involved in the metabolism of commonly used drugs. This raises the possibility of altered pharmacokinetics of medications such as anticoagulants, hormonal therapies, or central nervous system-active compounds [35,84,88,90].

Special attention should therefore be given to specific populations, including women with a history of hormone-dependent cancers, individuals receiving hormone replacement therapy, and patients undergoing long-term pharmacological treatment. In these groups, the use of hop-based supplements should be carefully evaluated, and further well-designed clinical studies are required to establish clear safety guidelines [27,35,83,84,86,87,89,90].

## 6. Biological Significance of Hop Bioactivity in the Context of Human Health

### 6.1. Cardiovascular System

Common hop represents a rich source of bioactive compounds exerting multidirectional effects on the cardiovascular system. Its biological activity includes cardioprotective, anti-atherosclerotic, antithrombotic, and hypotensive mechanisms [40], which result from the presence of numerous phytochemicals, particularly prenylated flavonoids (xanthohumol—XN, isoxanthohumol—IX, and 8-prenylnaringenin—8-PN), bitter acids (α- and β-acids), and polyphenols. These compounds affect lipid metabolism, endothelial function, platelet activity, and oxidative stress processes that underlie the development of cardiovascular diseases [40,84,92].

One of the best-documented aspects of hop activity is its influence on processes leading to the development of atherosclerosis. Xanthohumol shows a strong ability to inhibit triglyceride synthesis in hepatocytes (HepG2 cell line) and to markedly reduce apolipoprotein B secretion, which is of particular importance in the management of hypertriglyceridemia and lipoprotein disorders [93]. In animal studies conducted in models such as mice and rabbits, supplementation with xanthohumol resulted in reduced atherosclerotic plaque formation accompanied by improvement in the blood lipid profile [93,94]. Decreases in total cholesterol (TC) and LDL cholesterol levels, together with an increase in HDL cholesterol, were also observed, indicating a beneficial effect of these compounds on lipid balance [94].

In addition, xanthohumol acts as an inhibitor of cholesteryl ester transfer protein (CETP), thereby limiting cholesterol transport to lipoproteins that promote lipid deposition in the vascular wall. Another important anti-atherosclerotic mechanism is the ability of hop constituents to protect LDL particles against copper ion–induced oxidation, as oxidized LDL plays a key role in the initiation and progression of atherosclerotic lesions [93].

Hops and their derivatives also exhibit the ability to regulate blood pressure and protect vascular function. Bitter acids in the form of isohumulones, administered to patients with mild type 2 diabetes, not only improved glycemic control but also reduced systolic blood pressure, indicating their hypotensive potential [93]. Hop extract (HOP), xanthohumol and isoxanthohumol demonstrate a clear protective effect on blood vessels exposed to accelerated aging induced by particulate matter [95].

The mechanism underlying this effect is associated with a reduction in oxidative stress and inhibition of overexpression of markers of cellular and vascular aging, such as p53, p22phox, and the angiotensin II type 1 receptor (AT1R) [95]. Moreover, hop fractions exhibit vasorelaxant activity, as confirmed in ex vivo studies using isolated rat aortic rings, indicating a direct effect of these compounds on vascular smooth muscle tone [96].

An important area of hop activity is its influence on hemostasis and thrombotic processes. Xanthohumol demonstrates the ability to prevent both arterial and venous thrombosis without increasing the risk of bleeding, which represents a potential advantage over classical antiplatelet drugs such as aspirin. This mechanism involves inhibition of platelet activation induced by various agonists, including ADP and thrombin [97,98].

Xanthohumol reduces calcium ion release into the platelet cytoplasm and limits the expression of key platelet activation markers, such as P-selectin and integrin αIIbβ3. In addition, a polar lipid extract obtained from hops and beer shows strong anti-aggregatory activity against platelet-activating factor (PAF) and thrombin [98]. Furthermore, hop extract may enhance the anticoagulant properties of the endothelium by increasing the expression of ecto-ADPase (CD39), an enzyme responsible for the degradation of pro-aggregatory ADP [99].

Hop constituents also play an important role in protecting the myocardium against damage associated with ischemia and oxidative stress. Xanthohumol induces the expression of the Sirt1 protein, which contributes to the regulation of reactive oxygen species (ROS) levels and the prevention of mitochondrial dysfunction in both platelets and cardiomyocytes [98]. In ischemia–reperfusion (I/R) models, hop derivatives demonstrate the ability to modulate autophagy processes, thereby promoting cardiomyocyte survival under conditions of oxygen and nutrient deprivation [100].

Additionally, studies conducted in ovariectomized rats, a model of menopause, showed that administration of a mixture of clover and hop extracts led to improvement in vasorelaxation markers and reduction in oxidative stress levels. These findings suggest a potential role of hop constituents in reducing the risk of cardiovascular diseases in postmenopausal women [101].

Although most data regarding the effects of hops on the cardiovascular system originate from preclinical studies, promising results from human studies are also available. Supplementation with hop extract at a dose of 450 mg GAE/day for 60 days in healthy volunteers was safe and did not show toxicity in biochemical assessments [40,96]. Clinical studies have also confirmed that prenylated hop flavonoids are absorbed after oral administration and exhibit a linear dose–response relationship [23,93].

At the same time, caution is emphasized with regard to the use of high doses, as some hop flavonoids may interact with cytochrome P450 enzymes. This may lead to potential drug interactions, including with warfarin, which is of clinical relevance and requires further investigation [85].

### 6.2. Nervous System and Sedative Effects

Common hop exhibits multidirectional and complex effects on the nervous system, encompassing sedative, hypnotic, antidepressant, anxiolytic, and neuroprotective activities [102,103,104]. Its biological activity is primarily associated with the presence of bitter acids (α- and β-acids), essential oils, and flavonoids, particularly xanthohumol. Importantly, the mechanisms of hop action are not limited to a single molecular target but involve modulation of neurotransmitter receptors, effects on inflammatory processes within the brain, interactions with the autonomic nervous system, and components of the gut–brain axis [51].

With regard to sedative and sleep-promoting effects, hops have traditionally been used to alleviate insomnia, nervous excitability, and tension, which is reflected in the described neurobiological mechanisms [104,105,106]. Of particular importance is the modulation of GABA_A_ receptors, as inhibitory GABAergic neurotransmission represents one of the main calming systems in the central nervous system. Available data indicate that humulone acts as a positive allosteric modulator of GABA_A_ receptors, resulting in enhanced synaptic inhibition in the brain. This translates into specific functional effects, as humulone shortens sleep onset latency and prolongs sleep duration, thus influencing both sleep initiation and maintenance [106]. In addition, hop constituents may interact with adenosine receptors (A_1_ and A_2_), which are associated with relaxation and sedation, providing another biological pathway supporting the calming effect [102,107]. An important aspect of the practical use of hops is their synergy with valerian (Valeriana officinalis). The combination of these raw materials in the preparation Ze 91019 has been shown to exert central effects in humans, counteract caffeine-induced stimulation, and improve sleep quality, while notably showing no negative impact on cognitive performance the following day, which is relevant from a functional safety perspective [108].

Beyond effects on sleep and nervous tension, hops also show considerable potential in the modulation of mood and anxiety states, with clinical and preclinical data indicating anxiolytic and antidepressant properties [103,109]. The cited evidence highlights that supplementation with hop extract in healthy young adults resulted in a significant reduction in self-reported levels of anxiety, depression, and stress, as assessed using the DASS-21 scale, indicating an effect spanning multiple dimensions of psychological burden simultaneously [103]. Of particular interest is the mechanism attributed to matured hop bitter acids (MHBAs), whereby improvements in depressive-like behavior are associated with increased release of noradrenaline in the hippocampus and cerebral cortex, brain regions essential for the regulation of emotions, memory, and stress responses [110]. In this context, stimulation of the vagus nerve plays a key role and occurs through activation of bitter taste receptors (TAS2Rs) located in the gastrointestinal tract [110,111]. This suggests that the mood-modulating effects of hops may originate from gut-derived signaling, transmitted via the autonomic nervous system, ultimately leading to neurochemical changes in the brain. In addition, hops attenuate inflammation-induced depressive symptoms by reducing levels of pro-inflammatory cytokines in the brain, including IL-1β and TNF-α, which links their antidepressant effects to inhibition of neuroinflammation [109,110].

Another pillar of hop activity is its influence on cognitive functions and neuroprotection, understood as the ability to support mental performance and protect neurons from damage. The cited data indicate that iso-α-acids (IAAs) and MHBAs may enhance working memory, episodic memory, and executive functions, with the underlying mechanism involving activation of dopaminergic and noradrenergic systems—two key pathways regulating attention, motivation, cognitive control, and information processing [107,110,111,112,113]. From the perspective of structural brain changes, it has been reported that in older individuals, consumption of IAAs may be associated with increased gray matter volume, suggesting a potential effect counteracting age-related brain atrophy [114]. In parallel, cellular-level protective mechanisms have been emphasized. Xanthohumol and hop extracts activate the Nrf2/HO-1 pathway, which is associated with antioxidant responses and thereby increases the resistance of neuronal cells to oxidative stress and to cytotoxicity induced by glutamate or iron overload. In this context, neuroprotection is not merely a general strengthening effect but is linked to a specific pathway regulating cellular resilience [104,107,113]. Additionally, potential relevance has been indicated in the context of neurodegenerative diseases, as hop constituents may inhibit aggregation of amyloid-β and tau proteins, which are key processes associated with the development of Alzheimer’s disease. This places hops within the area of interest as a component of preventive strategies or supportive approaches in neurodegeneration [51,102,104,107,113].

The coherent integration of many of these effects is explained by the proposed gut–brain axis mechanism, which is particularly characteristic of hops, especially with regard to bitter acids [105,111]. Hop bitter compounds activate bitter taste receptors in the gut, present on enteroendocrine cells, leading to the release of cholecystokinin (CCK). This is followed by secondary activation of the vagus nerve, which transmits the signal to the central nervous system [110,111,112]. The signal reaches the locus coeruleus in the brainstem, resulting in modulation of neurotransmitter levels in higher brain structures. In practice, this mechanism serves as a bridge linking the activity of bitter compounds in the gastrointestinal tract with the observed central effects, ranging from regulation of mood and stress to support of cognitive functions [105,111,112].

### 6.3. Anticancer Potential

Common hop is a plant with an exceptionally rich phytochemical profile, whose constituents exhibit a broad spectrum of biological activities, including pronounced anticancer effects. Particular importance is attributed to prenylated flavonoids, especially xanthohumol (XN), and its structural derivatives such as tetrahydroxanthohumol (TXN), isoxanthohumol (IXN), and 8-prenylnaringenin (8-PN). These compounds are characterized by high biological reactivity, the ability to interact with multiple molecular targets, and relative selectivity toward cancer cells. Numerous in vitro and in vivo studies confirm that the anticancer activity of hops is pleiotropic in nature and involves the simultaneous modulation of cell proliferation, energy metabolism, inflammatory signaling, mechanisms of programmed cell death, and the invasive and metastatic potential of tumors [115,116,117].

One of the fundamental mechanisms underlying the anticancer activity of xanthohumol is the induction of programmed cell death, primarily in the form of apoptosis. This compound activates the caspase cascade, particularly caspase-3, leading to proteolytic degradation of the PARP protein and irreversible initiation of apoptotic pathways [117,118]. This process is closely associated with the induction of endoplasmic reticulum stress, manifested by increased expression of markers such as CHOP, ATF4, and IRE1α. Activation of these factors indicates disruption of protein homeostasis and overload of the protein-folding machinery, which in metabolically highly active cancer cells leads to surpassing the adaptive threshold and directing the cell toward apoptosis. This mechanism is particularly relevant for the elimination of cells resistant to classical apoptotic stimuli [117,118].

In parallel, xanthohumol (XN) and its derivatives, including tetrahydroxanthohumol (TXN), exhibit strong and unequivocal antiproliferative effects resulting from direct inhibition of cell cycle progression. Numerous experimental studies, conducted for example in human colorectal adenocarcinoma cells (HCT116 line), confirm induction of cell cycle arrest in the S and G2/M phases. This effect is closely linked to a significant reduction in the expression of key cell cycle regulators such as cyclin A and cyclin-dependent kinase CDK2, whose activity is essential for proper DNA replication and entry into mitosis. Effective blockade of these checkpoints leads to inhibition of further cell divisions, reduced proliferation, and gradual elimination of the cancer cell population. Importantly, these effects are observed at relatively low toxicity toward normal cells, which clearly highlights the high therapeutic and chemopreventive potential of hop-derived compounds [117,118].

Another important aspect of the anticancer action of xanthohumol is the modulation of key signaling pathways responsible for tumor survival, resistance, and aggressiveness. This compound effectively inhibits the activity of Akt kinase and the transcription factor NF-κB, which play central roles in regulating the expression of antiapoptotic, proinflammatory, and proliferation-promoting genes [119,120]. In addition, in multiple cancer models, including pancreatic ductal adenocarcinoma (PDAC), glioblastoma multiforme (GBM), and cholangiocarcinoma (CCA), suppression of the STAT3 and Notch1 pathways has been observed, leading to reduced angiogenesis, invasiveness, and maintenance of the cancer stem cell phenotype. Multidirectional inhibition of these pathways means that xanthohumol acts not only cytotoxically but also reprograms the oncogenic signaling network responsible for disease progression [121,122].

An additional, highly selective mechanism of xanthohumol action involves its effects on mitochondrial function in cancer cells. Xanthohumol inhibits the activity of complex I of the respiratory chain, leading to excessive production of reactive oxygen species and disruption of redox balance. Cancer cells, which already operate under conditions of elevated oxidative stress, are particularly sensitive to further increases in ROS levels, resulting in mitochondrial dysfunction, loss of mitochondrial membrane potential, and activation of the mitochondrial apoptotic pathway. This mechanism explains the selective toxicity of xanthohumol toward cancer cells with relative sparing of healthy cells [116,118,121].

In colorectal cancer (colorectal adenocarcinoma; SW-480 and HT-29 cell lines), xanthohumol and its derivatives exhibit particularly strong antiproliferative, anti-invasive, and chemopreventive effects. In addition to inducing cell cycle arrest in the S and G2/M phases, a marked reduction in the ability of cancer cells to migrate and invade tissues is observed. This effect is associated with inhibition of extracellular matrix metalloproteinases MMP-2 and MMP-9 and reduced expression of proangiogenic factors such as VEGF and HIF-1α [116,117,123]. Of particular importance is the effect of xanthohumol on colorectal cancer stem cells, which are responsible for disease recurrence and metastasis. Combination of xanthohumol with FOLFOX chemotherapy leads to suppression of the Wnt pathway and reduced expression of the CD44v6 marker, effectively limiting the metastatic potential of the tumor [116,124].

In pancreatic cancer (pancreatic ductal adenocarcinoma, PDAC), considered one of the most aggressive and treatment-resistant malignancies, xanthohumol shows especially promising properties. In studies using a transgenic KPC mouse model, a combination of xanthohumol with plumbagin resulted in significant prolongation of animal survival [122]. This mechanism involves simultaneous downregulation of antiapoptotic proteins such as BCL-2, inhibition of STAT3 phosphorylation and suppression of the Notch1 pathway, and reduced expression of survivin and cyclin D1. Such multidirectional activity enhances apoptosis, inhibits proliferation, and limits the adaptive capacity of pancreatic cancer cells [122].

In breast cancers (ER-positive estrogen-dependent tumors, MCF-7 cell line; HER2-positive/ErbB2-positive tumors; and triple-negative breast cancer, TNBC, e.g., MDA-MB-231 and Hs578T cell lines), hop-derived chalcones exhibit high cytotoxic selectivity, being several times more active against cancer cells than against normal epithelial cells [125]. Xanthohumol also plays an important role in the prevention of hormone-dependent cancers by acting as a strong aromatase inhibitor and limiting local estrogen synthesis, particularly in postmenopausal women. Moreover, this compound effectively overcomes multidrug resistance, sensitizing resistant breast cancer cell lines to doxorubicin and adriamycin [119,126].

In gliomas (including glioblastoma multiforme) and other central nervous system tumors (e.g., pilocytic astrocytoma and neuroblastoma), hop extracts disrupt cancer cell energy metabolism. Inhibition of glycolysis characteristic of the Warburg effect leads to reduced ATP and lactate production, depriving cancer cells of their primary energy sources. Simultaneously, inhibition of Akt phosphorylation, induction of oxidative stress, and modulation of microRNA expression, including increased levels of miR-204-3p, are observed, further promoting apoptosis and limiting glioma cell survival [120].

In leukemias (acute lymphoblastic leukemia, ALL; acute myeloid leukemia, AML; chronic myeloid leukemia, CML, including Bcr-Abl–positive forms such as the K562 line; chronic lymphocytic leukemia, CLL) and lymphomas (non-Hodgkin lymphoma, Burkitt’s lymphoma, B-cell lymphoma, multiple myeloma, and sarcomas such as osteosarcoma and fibrosarcoma, e.g., the HT-1080 line), including animal models in dogs, xanthohumol derivatives show high efficacy in inducing late apoptosis of cancer cells, particularly in populations characterized by high resistance to standard therapy [119,121,126,127,128]. This mechanism involves a marked reduction in the expression of antiapoptotic proteins from the Bcl-2 family, leading to destabilization of mitochondrial membrane integrity and facilitating cytochrome c release into the cytoplasm [117,119,122,127,128]. At the same time, enhanced generation of reactive oxygen species is observed, intensifying oxidative stress and exceeding the adaptive capacity of cancer cells, resulting in activation of the mitochondrial apoptotic pathway [117,118,121,128]. Particularly important is the ability of xanthohumol to increase the sensitivity of leukemia and lymphoma cells to classical cytostatics such as vincristine and cytarabine. This effect is especially pronounced in sequential treatment regimens in which xanthohumol is administered after chemotherapy, suggesting its role in overcoming adaptive mechanisms activated in cancer cells in response to cytotoxic stress [121,127,129,130].

The anticancer activity of xanthohumol has also been confirmed in a wide spectrum of solid tumors, including liver cancer (hepatocellular carcinoma, HCC; HepG2, Huh-7, Hep3B, and SK-Hep-1 cell lines), lung cancer (non-small cell lung cancer, including adenocarcinoma such as the A549 line and squamous cell carcinoma), melanoma, and cancers of the cervix (Ca Ski and HeLa cell lines), prostate (hormone-sensitive LNCaP and hormone-refractory PC-3 and DU145 lines), and thyroid (medullary thyroid carcinoma) [119,121]. Across all these models, a consistent molecular pattern is observed, including caspase-dependent apoptosis induction, PARP degradation, and inhibition of key survival pathways such as Akt, NF-κB, and Notch1 [117,118,119,121]. In addition, xanthohumol modulates inflammatory processes within the tumor microenvironment by reducing the expression of proinflammatory cytokines and angiogenic mediators, thereby limiting tumor growth and invasion of surrounding tissues [119,121,123]. Mitochondrial dysfunction, involving inhibition of the respiratory chain and increased oxidative stress, deepens the energetic deficits of cancer cells and enhances their susceptibility to apoptotic signals. Consequently, the action of xanthohumol is not limited to a cytotoxic effect but also includes attenuation of tumor biological aggressiveness and its adaptive and metastatic capacity [117,120,121,124].

An independent yet extremely important aspect of the anticancer activity of hops is their ability to enhance the efficacy of standard oncological therapies. Xanthohumol acts as a chemosensitizer by overcoming multidrug resistance mechanisms through inhibition of ABC transporters responsible for active efflux of cytostatics from cancer cells [119,121,124,125]. At the same time, it modulates key survival pathways activated in response to chemotherapy, such as Akt/mTOR, NF-κB, STAT3, and EGFR, thereby limiting the capacity of cancer cells to adapt and regenerate after treatment [115,119,121,124].

Of particular significance is the effect of xanthohumol on cancer stem cells, which are the main cause of relapse and metastasis. Combination with chemotherapy leads to inhibition of the Wnt pathway and reduced expression of markers of aggressive phenotype, effectively limiting tumor progression potential [124]. In addition, xanthohumol increases radiosensitization of cancer cells by enhancing apoptosis and autophagy induced by ionizing radiation. In clinical practice, these mechanisms may allow for reduction in cytostatic and radiation doses while maintaining high therapeutic efficacy and limiting treatment-related toxicity [119,123,124,129].

### 6.4. Dermatoprotective Properties of Hops and Their Bioactive Compounds

One of the key aspects of the dermatoprotective action of hops is their very strong antioxidant activity. Xanthohumol and lupulone belong to the most potent natural antioxidants [131,132,133,134], and the antioxidant activity of xanthohumol has been shown to surpass that of classical antioxidants such as vitamin C [132]. This mechanism is based on the effective scavenging of reactive oxygen species, including hydroxyl radicals, peroxyl radicals, superoxide anions, and the neutralization of singlet oxygen [131,133,135]. As a result, hop constituents protect skin cellular structures, particularly collagen, elastin, and the DNA of epidermal and dermal cells, against oxidative damage induced, among others, by UV radiation. This leads to a marked reduction in photoaging processes and premature degradation of skin tissues [132,133,136,137,138,139,140].

In parallel, hops exhibit strong anti-inflammatory activity, which plays a crucial role in the protection of both healthy skin and skin affected by pathological processes. Hop extracts effectively reduce the secretion of pro-inflammatory cytokines such as IL-6, IL-8, IL-1β, and TNF-α [131,133,137], and inhibit the activity of cyclooxygenase-2 (COX-2) and the synthesis of prostaglandins, particularly PGE_2_ [131,133,137,140]. In addition, blockade of MAPK and NF-κB signaling pathways limits the activation of inflammatory cascades at the cellular level. As a consequence, erythema, edema, pain, and skin irritation are reduced, which is of particular importance in the care of sensitive, reactive skin and skin affected by chronic dermatoses [137,141].

An important component of the dermatoprotective action of hops is also the protection of the skin’s extracellular matrix and anti-aging mechanisms. Hop constituents, especially xanthohumol and humulones, have the ability to inhibit the activity of proteolytic enzymes responsible for the degradation of skin supporting structures, such as elastase and matrix metalloproteinases (MMP-1, MMP-2, MMP-8, and MMP-9) [131,133,136,140,142]. At the same time, the synthesis of collagen types I, III, and V, elastin, and fibrillins in dermal fibroblasts is stimulated. The combination of these mechanisms leads to improved firmness, elasticity, and resilience of the skin, and a slowdown of skin aging processes [131,132,133,136,143,144].

Hops and their derivatives also exhibit pronounced antimicrobial activity, which is particularly important in protecting the skin against infections and microbiological disturbances. Lupulone and xanthohumol effectively inhibit the growth of Gram-positive bacteria, including skin pathogens such as Cutibacterium acnes and Staphylococcus aureus, also in the case of resistant strains such as MRSA [131,133,135,137,139,145,146]. In addition, antifungal activity against dermatophytes of the genus Trichophyton and antiviral activity, including against herpes simplex viruses HSV-1 and HSV-2, have been observed. Consequently, hops contribute to the stabilization of the skin microbiome and reduction in infection risk [131,132,143,147,148].

Particular attention should be paid to the ability of hops to reduce skin hyperpigmentation. Xanthohumol acts as a strong inhibitor of melanogenesis, both by directly inhibiting tyrosinase activity and by limiting the transport of melanosomes from melanocytes to keratinocytes [131,132,133,136,139,140,149]. In addition, an effect on the degradation of already accumulated melanin in skin cells has been observed. This dual mechanism of action makes hop constituents effective in lightening hyperpigmentation and evening out skin tone [131,133,136,139].

These properties find direct application in the prevention and supportive treatment of skin diseases. In acne vulgaris, hops act in a multidirectional manner, combining antibacterial effects against C. acnes with strong anti-inflammatory, antioxidant, and sebum-regulating activity [69,131,132,133,135,137,150]. In psoriasis and atopic dermatitis, hop extracts alleviate inflammation, limit excessive proliferation of epidermal cells, and support the restoration of the lipid barrier [131,133,137,139]. Particularly important is also the wound-healing activity, in which CO_2_ extracts of hops stimulate granulation tissue formation, cleanse wounds from necrotic tissue, and normalize the skin microbiota, including in infected wounds [132,133,150,151]. In the scalp area, hops exhibit anti-dandruff, antifungal, and anti-seborrheic effects and strengthen hair follicles, reducing hair loss and brittleness [132,135,143].

The use of hops in cosmetics covers a wide range of dosage forms. The most commonly used are creams and emulsions, particularly oil-in-water (O/W) types, in which better release of active substances is observed compared to W/O formulations [137]. The typical concentration of hop extract in such preparations is approximately 1%. In gels and facial cleansing products, lower concentrations, around 0.3%, are used, allowing effective care of acne-prone skin without causing irritation [69,131]. Hops are also used in hair care products and deodorants, where they limit the growth of bacteria responsible for unpleasant body odor, and are a natural preservative component in cosmetic formulations [131,132,135,139,143,152]. An additional form of application is beer baths, which improve skin hydration, skin tone, and regulate keratinization processes [135] (Table 1).

## 7. Discussion

The data presented in this study clearly indicate that the biological activity of hops (*Humulus lupulus* L.) is multidirectional and results from the synergistic action of numerous groups of bioactive compounds rather than from a single constituent. Bitter acids, prenylated flavonoids, and essential oils interact with common and partially overlapping molecular pathways, which makes it difficult to unambiguously assess their individual contributions to the observed biological effects. This phenomenon highlights the validity of analyzing whole hop extracts rather than isolated compounds, particularly in the context of potential functional applications. At the same time, this complexity makes standardization of extracts and comparison between studies challenging, as differences in composition, extraction methods, and dosing strategies may significantly influence biological outcomes.

A significant interpretative challenge remains the limited bioavailability of key prenylflavonoids, especially xanthohumol and 8-prenylnaringenin. Despite their strong activity demonstrated in in vitro studies and preclinical models, their extensive metabolism and low solubility markedly limit the concentrations achieved in systemic circulation. At the same time, the involvement of the gut microbiota in the biotransformation of isoxanthohumol into 8-prenylnaringenin points to substantial interindividual variability in biological effects, which may explain the discrepancies observed between experimental and clinical study outcomes. Moreover, many experimental studies employ concentrations that are difficult to achieve under physiological conditions, which may lead to overestimation of the actual in vivo efficacy of these compounds.

The significance of the estrogen-like activity of hops in terms of the balance between benefits and risks also remains a matter of debate. On the one hand, the efficacy of 8-prenylnaringenin in alleviating menopausal symptoms and its beneficial effects on bone metabolism have been documented; on the other hand, its high estrogenic potency raises concerns regarding the safety of long-term supplementation, particularly in the absence of standardized preparations. This underscores the need for cautious interpretation of research findings and for further clinical studies aimed at defining safe dosage ranges. In addition, potential interactions with hormone-dependent conditions and medications should be carefully considered, as current evidence in humans remains limited and sometimes inconsistent.

In the context of civilization-related diseases, observations concerning the modulation of metabolic and inflammatory processes by hop-derived compounds are of particular interest. Available data suggest that hops may simultaneously influence insulin resistance, lipid metabolism, and chronic inflammation, which aligns with current concepts of multifactorial prevention of metabolic syndrome. It should be emphasized, however, that most of the available evidence originates from preclinical studies, and its direct translation into clinical practice requires further validation. The relatively small number of well-designed, randomized clinical trials, together with heterogeneity in study protocols, further limits the strength of current conclusions and highlights the need for standardized human studies.

## 8. Conclusions

Common hop (*Humulus lupulus* L.) is a plant raw material with an exceptionally broad and multi-level biological potential, resulting from the presence of numerous secondary metabolites with diverse mechanisms of action. Bitter acids, prenylated flavonoids, and essential oils form a functionally interconnected bioactive system in which additive and synergistic effects are of key importance, rather than the action of individual compounds. For this reason, hops should be regarded as a complex functional raw material rather than merely a source of isolated active substances.

The collected data indicate that the antioxidant, anti-inflammatory, metabolic, and hormonal mechanisms of hop activity are closely interrelated and may jointly influence processes underlying civilization-related diseases, such as chronic inflammation, insulin resistance, lipid disorders, and oxidative stress. Of particular importance is the ability of hop-derived compounds to modulate key signaling pathways, including NF-κB, Nrf2, MAPK, AMPK, and nuclear PPAR receptors, which confirms their pleiotropic biological nature.

At the same time, a major limitation of the practical use of hops remains the low bioavailability of the most important prenylflavonoids and the high interindividual variability of biological responses, which depends, among other factors, on the composition of the gut microbiota. This means that the high activity observed in in vitro and preclinical studies does not always translate directly into in vivo effects, necessitating cautious interpretation of results and further pharmacokinetic and clinical investigations.

Of particular relevance is the estrogen-like activity of hops, which on the one hand underlies their potential use in alleviating menopausal symptoms and protecting bone tissue, but on the other raises important safety concerns. The high estrogenic potency of 8-prenylnaringenin highlights the need for precise standardization of hop preparations and careful dose control, especially in the context of long-term use and possible hormonal interactions.

## Figures and Tables

**Table 1:** Summary of biological activities and molecular targets of hop-derived compounds.

Compound	Pathway	Mechanism	Effect	Study Type
Xanthohumol (XN)	NF-κB, Nrf2	inhibition/activation	anti-inflammatory, antioxidant	in vitro/in vivo
8-prenylnaringenin (8-PN)	ERα, ERβ	agonist	estrogenic	in vivo/clinical
Isoxanthohumol	microbiota-dependent	biotransformation → 8-PN	indirect estrogenic	in vivo
Bitter acids (α, β)	AMPK, PPAR	activation	metabolic regulation	animal
Polyphenols	ROS, AMPK	scavenging/modulation	antioxidant	in vitro

## Data Availability

Data sharing does not apply in this article as no datasets have been generated.
